# LOC102549726/miR-760-3p network is involved in the progression of ISO-induced pathological cardiomyocyte hypertrophy via endoplasmic reticulum stress

**DOI:** 10.1007/s10735-023-10166-1

**Published:** 2023-10-30

**Authors:** Bangsheng Chen, Lian Tan, Ying Wang, Lei Yang, Jiequan Liu, Danqi Chen, Shuaishuai Huang, Feiyan Mao, Jiangfang Lian

**Affiliations:** 1 Emergency Medical Center, Ningbo Yinzhou No. 2 Hospital, Ningbo, Zhejiang 315192 China; 2 Intensive Care Unit, Ningbo Yinzhou No. 2 Hospital, Ningbo, Zhejiang 315192 China; 3 Cadiovascular Department, Ningbo Medical Center LiHuiLi Hospital, Ningbo, Zhejiang 315100 China; 4 Laboratory of Renal Carcinoma, Ningbo Yinzhou No. 2 Hospital, Ningbo, Zhejiang 315192 China; 5 Department of General Surgery, HwaMei Hospital, University of Chinese Academy of Sciences, Ningbo, Zhejiang 315100 China

**Keywords:** Neonatal rat cardiomyocytes, Cardiac hypertrophy, LOC102549726, miR-760-3p, ER stress

## Abstract

Pathological cardiac hypertrophy (CH) is featured by myocyte enlargement and cardiac malfunction. Multiple signaling pathways have been implicated in diverse pathological and physiological processes in CH. However, the function of LOC102549726/miR-760-3p network in CH remains unclear. Here, we characterize the functional role of LOC102549726/miR-760-3p network in CH and delineate the underlying mechanism. The expression of LncRNA LOC102549726 and hypertrophic markers was significantly increased compared to the control, while the level of miR-760-3p was decreased. Next, we examined ER stress response in a hypertrophic cardiomyocyte model. The expression of ER stress markers was greatly enhanced after incubation with ISO. The hypertrophic reaction, ER stress response, and increased potassium and calcium ion channels were alleviated by genetic downregulation of LOC102549726. It has been demonstrated that LOC102549726 functions as a competitive endogenous RNA (ceRNA) of miR-760-3p. Overexpression of miR-760-3p decreased cell surface area and substantially mitigated ER stress response; protein levels of potassium and calcium channels were also significantly up-regulated compared to the NC control. In contrast, miR-760-3p inhibition increased cell size, aggravated CH and ER stress responses, and reduced ion channels. Collectively, in this study we demonstrated that the LOC102549726/miR-760-3p network was a crucial regulator of CH development. Ion channels mediate the ER stress response and may be a downstream sensor of the LOC102549726/miR-760-3p network. Therefore, these findings advance our understanding of pathological CH and provide new insights into therapeutic targets for cardiac remodeling.

## Introduction

Cardiac hypertrophy (CH) and subsequent heart failure are the leading causes of death worldwide (Rosenbaum et al. 2020). Initially, CH is an adaptive response that maintains cardiac output. However, prolonged CH can ultimately impair heart function, leading to heart failure and death (Xiang et al. 2021). Given that CH plays a vital role in the progression of cardiovascular diseases, it is essential to identify the molecular mechanism underlying the prevention and treatment of pathological CH.

The endoplasmic reticulum (ER) plays an important role in many cellular physiological processes (Oakes and Papa 2015). Numerous extracellular stimuli, including gene mutation, calcium deficiency, ischemia, free radical exposure, and increased protein synthesis, can disrupt ER homeostasis and contribute to ER stress (ERS) (Rani et al. 2017; Shen et al. 2020). ERS activates a cytoprotective reaction called unfolded protein response (UPR) signaling pathway (Das et al. 2021). In mammalian cells, the UPR signaling pathway consists of three main sensors: protein kinase R (PKR)-like ER kinase (PERK), activating transcription factor 6 (ATF6), and endoribonuclease inositol requiring enzyme 1α (IRE1α) (Hetz et al. 2020). Pathological CH is closely associated with ERS, resulting in maladaptation, including myocardial fibrosis, cardiac dysfunction, and induction of apoptosis (Li et al. 2015; Zhang et al. 2019).

As highly conserved, small non-coding RNAs (21—24 nucleotides), microRNAs (miRNAs) have been validated as essential regulators of cell differentiation, proliferation, and apoptosis (Correia de Sousa et al. 2019). They exert their activity by binding to the complementary 3’ untranslated region, thereby inhibiting the expression of target mRNAs at the post-transcriptional level (Omidkhoda et al. 2019). Numerous miRNAs have been identified as important regulators in CH and heart failure pathophysiology. For example, miR-378 positively regulates CH through the mitogen-activated protein kinase (MAPK) signaling pathway (Zhou et al. 2021), miRNA30a induces left ventricular hypertrophy, and miRNA489 protects against CH (Pan et al. 2013; Wang et al. 2014).

Long non-coding RNAs (lncRNAs) are a cluster of transcribed RNA molecules longer than 200 nucleotides that lack protein-coding potential (Zhang et al. 2018). Recent studies have demonstrated that lncRNAs emerge as key regulators in various disease processes, including pathological CH (Han et al. 2017; Lu et al. 2022). For instance, lncRNA Myosin heavy chain associated RNA transcript (Mhrt) has been depicted to protect the heart against pathological CH (Zhang et al. 2020a). LncRNA H19 has been validated as a negative regulator of CH, and CH-associated epigenetic regulator (Chaer) is necessary for the development of CH (Liu et al. 2016; Viereck et al. 2020; Wang et al. 2016). Additionally, lncRNAs regulate the transformation of cellular signals by various mechanisms, including RNA processing, apoptosis modulation, and chromatin modification (Xiao et al. 2019). They also serve as competing endogenous RNA (ceRNA) (Rinn and Chang 2012). CeRNA has been proposed as a novel regulator of non-coding RNA and coding RNA. Generally, lncRNAs can act as ceRNAs by competitively interacting with target miRNAs through a sponge-like action. This interaction leads to the release of target mRNAs from repression by the targeted miRNAs, thereby influencing and regulating the expression of target genes (Xiao et al. 2019). It has been demonstrated that CH-related factor (CHRF) regulates CH by targeting miR-489 (Wang et al. 2014). LncRNA UCA1 was validated as a positive regulator in hypertrophy response of cardiomyocytes via sponging miR184 (Zhou et al. 2018). Recent studies have improved our understanding of CH progression. However, our knowledge of the biological mechanisms underlying lncRNA/miRNA networks remains limited.

In the previous investigations, the co-expression patterns of lncRNA and miRNA in ceRNA have been well established (Tay et al. 2014). Moreover, the LOC102549726/miR-760-3p network has been previously confirmed (E Q et al. 2020). However, no additional research has been conducted regarding the role of the LOC102549726/miR-760-3p network in CH development. This study aimed to explore the functional role of the LOC102549726/miR-760-3p network in CH, decipher the underlying mechanisms, and identify new therapeutic targets for pathological CH.

## Materials and methods

### Primary cell isolation and treatment

All animal experiments complied with the Animal Management Guidelines of the Chinese Ministry of Health and were approved by the Institutional Animal Care and Use Committee of Ningbo University. Male Sprague-Dawley rats were purchased from the Animal Experimental Center of Ningbo University. All animals were maintained under standard conditions. Primary neonatal rat cardiomyocytes (NRCMs) were isolated from the hearts of one- to three-day-old rats. Briefly, after removing atria, ventricles from 20 hearts were dissected and dissociated in Dulbecco’s Phosphate-Buffered Saline (D-PBS, Solarbio, Beijing, China), after which the fibroblasts and vessels were removed. Subsequently, ventricles were trimmed into blocks with about 1 mm in diameter. These blocks were then digested with 0.25% trypsin and 0.2% collagenase type II (Solarbio, Beijing, China). The digestion was halted by addition of serum-containing medium. The resulting cell suspension was centrifuged at 1500 × g for 5 min, following which the supernatant was discarded and the NRCMs were isolated. NRCMs were plated into six-well culture plates at a density of 1 × 10^6^ cells per well and incubated in complete Dulbecco’s modified Eagle’s medium (DMEM, Hyclone, UT, USA) supplemented with 10% fetal bovine serum, 1% penicillin, and streptomycin in humidified air with 5% CO_2_ at 37 °C. Cells were collected at zero, eight, 18, and 24 h after exposure to 10 μmol/L of isoproterenol (ISO).

### Plasmids and miRNA transfection

miR-760-3p mimic, negative control miRNA (miR-NC), miR-760-3p inhibitor, and miRNA inhibitor negative control (anti‐miR‐NC) were synthesized by Sangon Biotech (Shanghai, China). LOC102549726 small interference RNA (siRNA) and a siRNA negative control (si-NC) fragment were purchased from General Biology (Ahhui, China). NRCMs were seeded in 6‐well culture dishes to reach a density of 70% and 50 nm of siRNA, miRNA mimics, and miRNA inhibitors were transfected using 5 μl of Lipofectamine^®^ 3000 Transfection Reagent (Thermo Fisher Scientifc, Waltham, MA, USA) with 125ul of Opti-MEM^®^ (Invitrogen), according to the manufacturer’s protocol. Six h post-transfection, the supernatant in the cell culture was discarded and fresh medium was replenished. Experiments were conducted 48 h post-transfection, or treated with 10 μmol/L of ISO for another 24 h prior to subsequent experiments. The sequences of miRNA and siRNA used are listed in Table 1.

**Table 1:** Sequences of miRNA and siRNA used to transfection

miR-760-3p mimic	sense	CGGCUCUGGGUCUGUGGGGA
	antisense	UCCCCACAGACCCAGAGCCG
mimic NC	sense	UCACAACCUCCUAGAAAGAGUAGA
	antisense	UCUACUCUUUCUAGGAGGUUGUGA
miR-760-3p inhibitor	sense	UCCCCACAGACCCAGAGCCG
inhibitor NC	sense	UCUACUCUUUCUAGGAGGUUGUGA
siR-LOC102549726-677	sense	(+ A)(+ A)(+ A)CAGATACACTTCA(+ G)(+ G)(+ C)
siR-LOC102549726-435	sense	(+ T)(+ T)(+ A)GACAATGAGATGA(+ T)(+ C)(+ A)
siR-LOC102549726-729	sense	(+ T)(+ T)(+ A)TTAGAAGCAGGAA(+ A)(+ C)(+ C)
siR-NC	sense	UUCUCCGAACGUGUCACGUTT
	antisense	ACGUGACACGUUCGGAGAATT

“+” indicates LNA (Locked Nucleic Acid) modifications

### Immunofluorescence (IF) staining

Immunofluorescence staining was performed according to the manufacturer’s instructions (proteintech, Wuhan, China). Briefly, the cultured NRCMs were fixed with 4% paraformaldehyde for 15 min, followed by adding 0.05% Triton X-100 for an additional 15 min. After 30 min of blocking with 5% bovine serum albumin (BSA) at 37 °C, the cells were incubated with diluted primary antibody α-actin (1:100; cat. no. 23660-1-AP; Proteintech) overnight at 4 °C. The cells were incubated with Cy3 goat anti-rabbit IgG (1:200; cat. no. As007; ABclonal) for 30 min at 37 °C The nuclei were stained with DAPI (KGA215-50; KeyGEN BioTECH Co., Ltd.) for 5 min in the dark and then sealed with 50% glycerol. Fluorescent signals were detected using a fluorescence microscope (CKX53; OLYMPUS; magnification, x400). ImageJ software (v1.37, National Institutes of Health) was used for quantification. Blue fluorescence indicates the nucleus, and red fluorescence indicates α-actin.

### Histological assay

NRCMs were fixed with 4% paraformaldehyde for 15 min, washed three times with PBS at 5 min intervals, and stained with hematoxylin and eosin (H&E). NRCMs were visualized under a microscope (Nikon 80i).

### Cell viability assay

Cell viability was evaluated using the Cell Counting Kit-8 (CCK-8). Briefly, each batch of NRCMs was seeded in a 96-well plate at approximately 2 × 10^4^ cells per well. Next, 10 μL of CCK-8 (KGA317; KeyGEN BioTECH Co., Ltd.) was added to each well and incubated for 2 h. The absorbance of each well was measured at 450 nm using a microplate reader (WD-2102B; Beijing LiuYi Biotechnology CO., LTD.). Cell viability was calculated using the data acquired above.

### Quantitative real-time PCR(qRT-PCR)

Total RNA was extracted from NRCMs using TRIzol reagent (CW0580S, CWBIO) according to the manufacturer’s instructions. RNA was reverse-transcribed into cDNA using oligo-dT and the Superscript First-strand Synthesis System (MR101-02; Vazyme). The relative expression of specific RNA was detected using an ABI 7500 real-time PCR system with SYBR Green (MQ101-02; Vazyme). β-actin was used as an internal control for mRNA expression. U6 was used as an internal control for miRNA quantification. The nucleotide sequences of primers used are listed in Table 2.

**Table 2:** Primer sequences and product size (base pair = bp)

Gene	Sequence		Product length (bp)
β-actin	Forward	GCCATGTACGTAGCCATCCA	375
β-actin	Reverse	GAACCGCTCATTGCCGATAG	
ANP	Forward	GGGCTTCTTCCTCTTCCTG	248
ANP	Reverse	TCTGAGACGGGTTGACTTCC	
BNP	Forward	TCCTGCTTTTCCTTAATCTGTC	254
BNP	Reverse	GCTTGAACTATGTGCCATCTTG	
β-MHC	Forward	CAGGAAGAACCTACTGCGGC	232
β-MHC	Reverse	CTCATTCAGGCCCTTGGCAC	
LOC102549726	Forward	ACAATGAATGGGCTTTGCCG	270
LOC102549726	Reverse	TCGGATAGCTGGCAACAAGG	
ATF6	Forward	ACACAGAAACCACTAGCATCAGTA	179
ATF6	Reverse	TGTGGTCTTGTTATGGGTGGTA	
miR-760-3p	Forward	CGCGGCTCTGGGTCTG	64
miR-760-3p	Reverse	AGTGCAGGGTCCGAGGTATT	
U6	Forward	ATTGGAACGATACAGAGAAGATT	90
U6	Reverse	GGAACGCTTCACGAATTTG	

### Western blotting

Total protein was extracted from cultivated cardiac myocytes by RIPA lysis buffer (C1053, APPLYGEN, Beijing, China) supplemented with a mixture of protease and phosphatase inhibitor cocktail (GRF102, Epizyme, Shanghai, China). Lysates were centrifuged at 12,000 × g for 20 min at 4 °C. The supernatant was collected, and the total protein content was quantifed using the BCA Protein Assay Kit (E-BC-K318-M, Elabscience, Wuhan, China). Protein samples (30 μg) were separated using sodium dodecyl sulfate-polyacrylamide gel electrophoresis (6–12%) and then transferred to a polyvinylidene difluoride (PVDF) membrane. The membranes were blocked with 5% skimmed milk in Tris-buffered saline Tween (TBST) for 1 h at room temperature and then incubated with the following primary antibodies; Rabbit anti rat ATF6 (1:1000; cat. no. ab37149; Abcam), Rabbit Anti rat p-IRE1 (1:1000; cat. no.14C10; CST), Rabbit Anti rat p-eIF2α (1:1000; cat. no. #9721; CST), Rabbit Anti rat XBP-1 (1:1000; cat. no. abs115725; absin), Rabbit Anti rat ATF4 (1:1000; cat. no. abs135528; absin), Rabbit Anti rat HERG (1:1000; cat. no. APC-062; alomone labs), and Rabbit Anti rat SERCA2 (1:1000; cat. no. 4388; CST) overnight at 4 °C. After 2 h of incubation with HRP-conjugated secondary antibodies supplemented with enhanced chemiluminescence (ECL) reagents (RJ239676; Invitrogen), immunoreactive protein bands were visualized using a chemiluminescent imaging system (Tanon-5200, Shanghai Tanon Biotechnology CO., LTD.).

### Imaging and image analysis

#### Immunofluorescence imaging

The purity of NRCMs was assessed by immunofluorescent staining for α-actin. Briefly, α-actin was used to stain NRCMs, and nuclei was stained with DAPI. The presence of α-actin in the cytoplasm of NRCMs could be visualized by red fluorescence, while the DAPI stained nuclei could be visualized in blue fluorescence. Cellular impurities in the cells did not show red fluorescence, only the nuclei were stained blue. The purity of NRCMs was calculated by dividing the number of α-actin positive cells by the total number of DAPI positive nuclei, after counting five different fields.

#### Analysis of cardiomyocyte size

Histology images were analyzed using Image J software. Briefly, the images were exported as TIFF files and converted to 8-bit grayscale. Thereafter, the “Find Edges” and “Threshold” processes were applied to obtain the cell perimeter. Subsequently, the perimeter was selected and the area was measured using the “Analyze Particles” function with the “Include Holes” option selected. Experimental values were presented as mean ± SD. GraphPad Prism 8.0 software was used to generate bar graphs based on the values aquired above.

#### Analysis of western blot

Blot bands were quantified using Image J software through densitometry. The bands were visualized using the chemiluminescence method, and the overall gray values of the protein bands (average gray value area) were quantified. Additionally, GAPDH was used as a loading control to compare the gray value of target protein in different groups.

### Statistical analysis

Data are presented as means ± standard deviation (SD). Student’s t-test and one-way analysis of variance (ANOVA) were performed to evaluate statistical differences in the mean values. Three independent experiments were conducted throughout the study, unless otherwise specified. Statistical significance was set at p < 0.05. SPSS software (version 20.0, SPSS Inc., Chicago, IL) was used to perform statistical analyses.

## Results

### Establishment of cardiac hypertrophy model in vitro

**Fig. 1:**
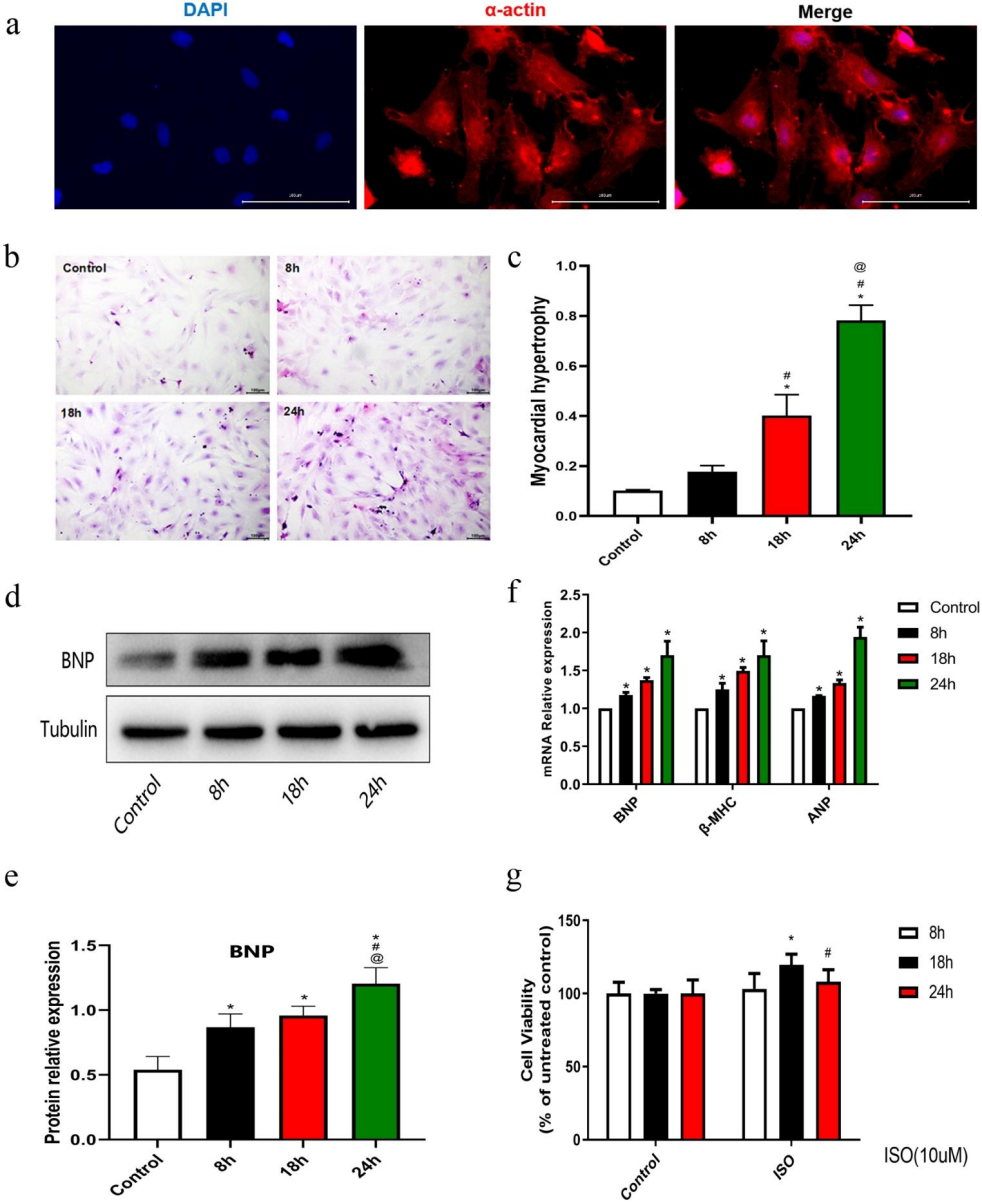
The establishment of primary neonatal rat cardiomyocyte hypertrophy (NRCM) model by ISO treatment. (**a**) Morphaology and identification of NRCMs (400X). α-actin (Red) was used to stain NRCMs, and nuclei was stained with DAPI (blue), merged as indicated in the upper row. The positive rate of α-actin reached 92.9%. n = 5. (**b,c**) Representative H&E staining images and quantitative analyses of myocardial hypertrophy relative to NRCMs with or without ISO treatment based on cell surface area. The cells enlarged proportional to the ISO incubation time. The datas are represented as mean ± SD, n = 3 for each group. Scale bar = 100 μm. ^*^p < 0.05 vs. control group; ^#^p < 0.05 vs. ISO 8-h group; ^@^p < 0.05 vs. ISO 18 h group, respectively using Student’s t-test and one way ANOVA test. (**d,e**) Representative immunoblots and quantitative analyses of hypertrophic marker BNP in the indicated groups. ISO-induced expression of BNP was markedly up-regulated in the indicated times. n = 3 in each group, Bars show the mean ± SD of the band density normalized to the tubulin. ^*^p < 0.05 as compared to control group; ^#^p < 0.05 vs. ISO 8-h group; ^@^p < 0.05 vs. ISO 18 h group, using Student’s t-test and one way ANOVA test. (**f**) The relative mRNA expression levels of the cardiomyocyte hypertrophic markers BNP, β-MHC, and ANP in NRCMs after treatment with PBS and ISO at different time points. ISO-induced expression of BNP, β‐MHC, and ANP was markedly up-regulated in the indicated times. Bars show the mean values ± SD. n = 3, ^*^p < 0.05, vs. control group using an ANOVA test. (**g**) The CCK-8 assay evaluated the levels of cell viability in response to ISO incubation. Bars show the mean values ± SD. n = 3, ^*^p < 0.05 vs. ISO 8-h group; ^#^p < 0.05 vs. ISO 18 h group, respectively using Student’s t-test

To establish a hypertrophic cardiomyocyte model, we examined the cellular state of NRCMs using immunofluorescence staining. Results revealed that cardiomyocytes exhibited irregular shapes such as rhomboids and polygons (Fig. 1A). The purity of α-actin reached 92.9%, proving that primary NRCMs were successfully isolated. NRCMs were then treated with the recommended ISO dosage at different time points, as described above. Hematoxylin and eosin (H&E) staining of NRCMs revealed that myocardial hypertrophy was proportional to the ISO incubation time, as evidenced by the increased cell surface area (Fig. 1B and C). The mRNA levels of hypertrophic markers, including atrial natriuretic peptide (ANP), brain natriuretic peptide (BNP), and β-myosin heavy chain (β-MHC) were measured to demonstrate the efficiency of the established cardiomyocyte hypertrophy model. ISO-induced expression of ANP, BNP, and β-MHC was markedly up-regulated in the indicated times (Fig. 1F). Furthermore, we detected the protein expression level of BNP which has a similar tendency with mRNA, peak at 24 h (Fig. 1D and E). Additionally, the CCK-8 assay was used to evaluate the cell viability at different incubation times. There were no differences between the control group at different time points; however, compared to the 8-h incubation group, the 18-h incubation with ISO significantly enhanced cell viability. Interestingly, the enhancement was attenuated to almost the basal level at 24 h (Fig. 1G).

### Expression of LOC102549726/miR-760-3p and ER stress sensors in pathological cardiac hypertrophy

**Fig. 2:**
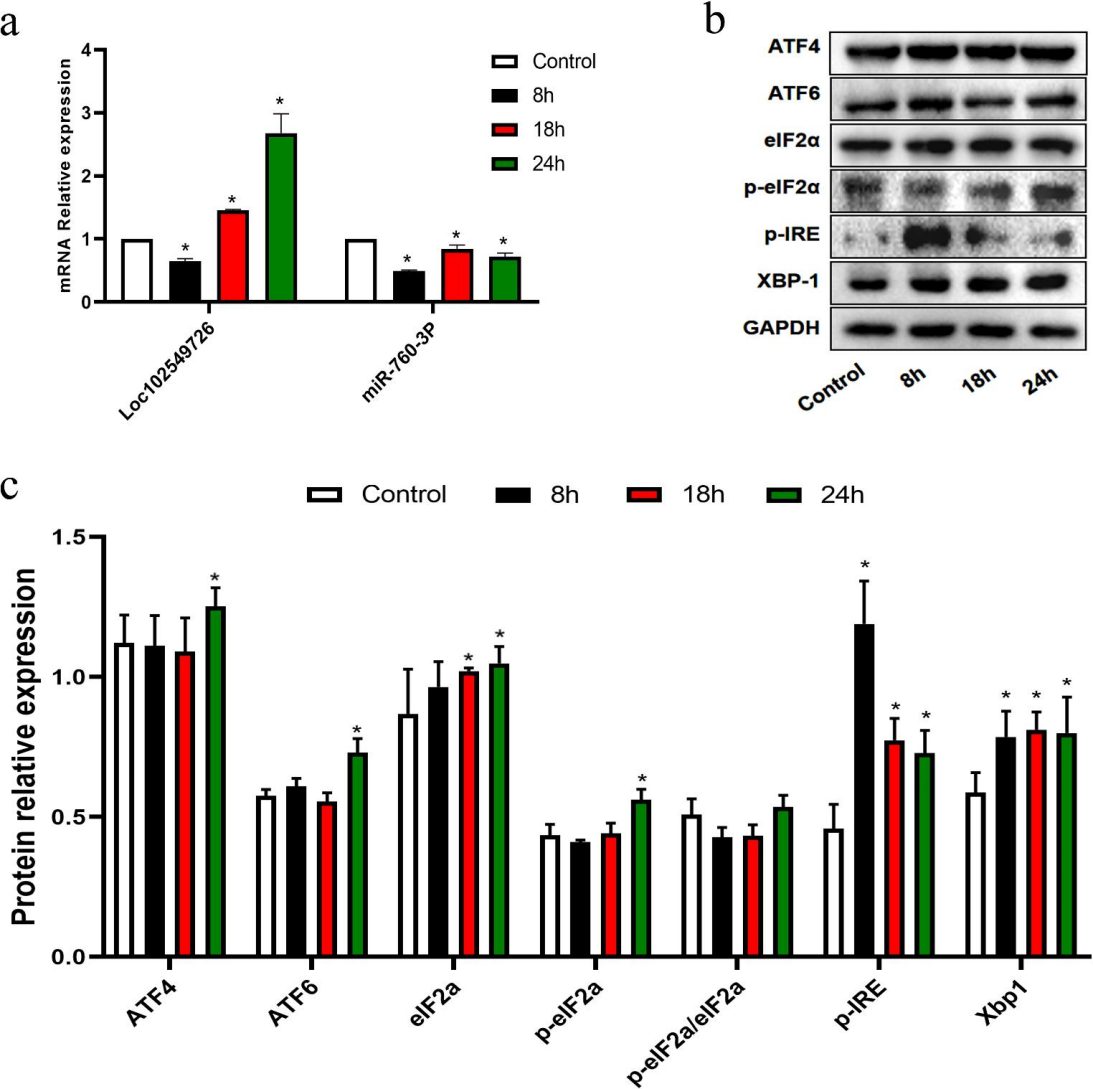
The expression of LOC102549726/miR-760-3p and ER stress sensors were altered in pathological CH. (**a**) Detection of LOC102549726 and miR-760-3p levels in NRCMs from PBS and ISO treatment groups by real-time PCR. The level of LOC102549726 increased at 18 and 24 h, whereas the expression of miR-760-3p was significantly decreased in all ISO-treated groups compared with that in the control group. Bars show the mean values ± SD. n = 3, ^*^p < 0.05, vs. control group, using an ANOVA test. (**b,c**) Representative immunoblots and quantitative analyses of ER stress sensors ATF4, ATF6, eIF2α, p-eIF2α, p-IRE, and XBP-1 from the indicated groups. p-eIF2α, ATF4 and ATF6 protein levels were found increased after incubation with ISO for 24 h, whereas the levels of p-IRE and XBP-1 were significantly increased in all ISO-treated groups compared to the control. Values are mean ± SD. n = 3 in each group. ^*^p < 0.05 vs. control group, using an ANOVA test

To determine whether LOC102549726/miR-760-3p levels were altered in CH, we measured the LOC102549726 and miR-760-3p levels in ISO-treated cardiomyocytes. As illustrated in Fig. 2A, 8 h of ISO incubation decreased the mRNA level of LOC102549726 relative to that in the control group, but 18- and 24-h incubation resulted in a significant increase. The mRNA level of miR-760-3p was significantly decreased in all ISO-treated groups compared with that in the control group. Additionally, the protein levels of ER stress-associated molecules in CH were determined. Our results depict that the relative expression of p-eIF2α/eIF2α in ISO-treated cardiomyocytes remained unaltered. Moreover, p-eIF2α, ATF4 and ATF6 were found to be up-regulated after incubation with ISO for 24 h, although there was no significant difference after eight or 18 h. Furthermore, the protein levels of p-IRE and XBP-1 were significantly increased in all ISO-treated groups compared to the control (Fig. 2B and C). Based on the above results, we selected a 24 h ISO incubation time for subsequent experiments.

### Knockdown of LOC102549726 attenuates hypertrophic response in ISO-induced cardiac hypertrophy

**Fig. 3:**
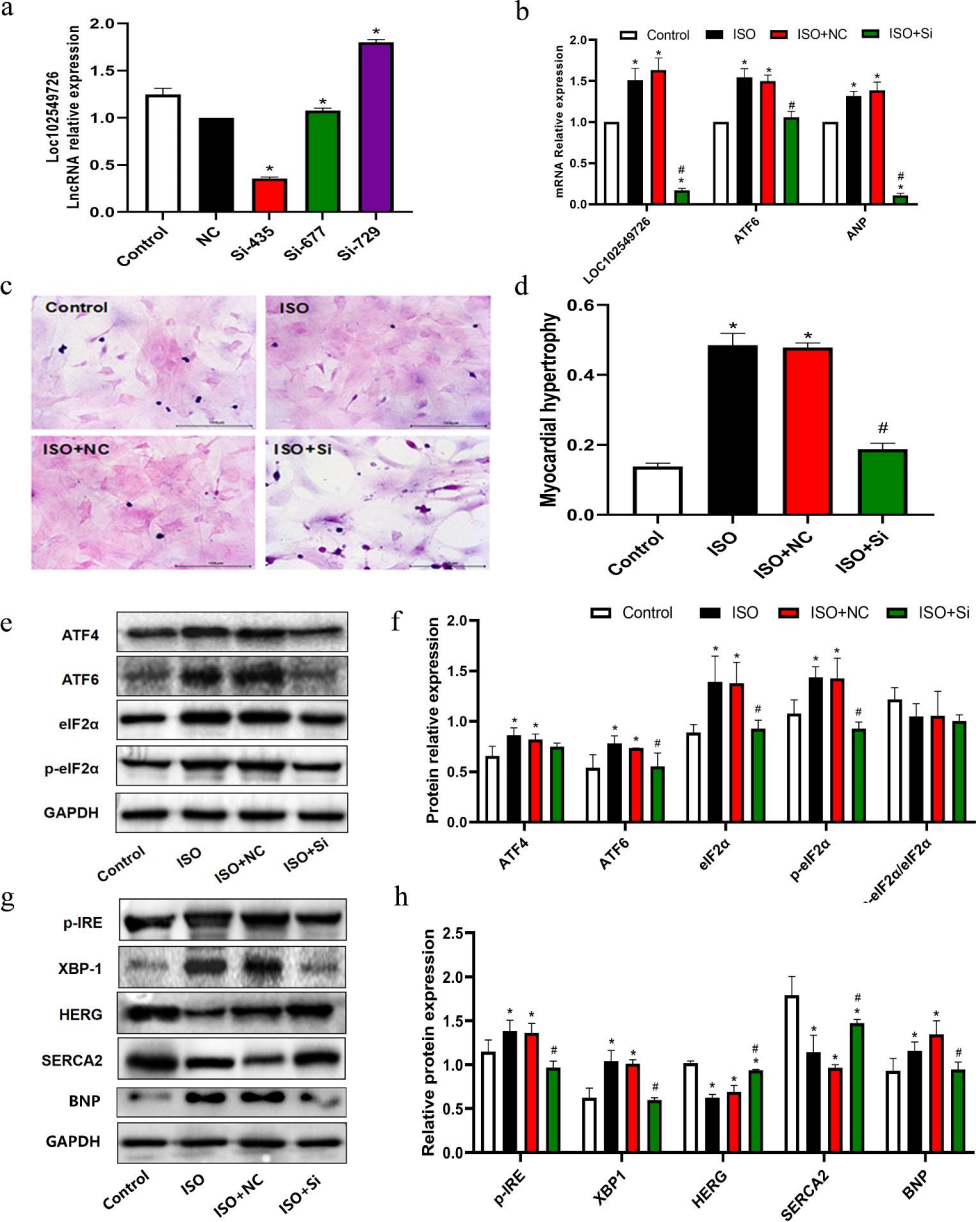
LOC102549726 promotes CH via ER stress in ISO-treated NRCMs. (**a**) The interfering effect of the three synthesized siRNA-LOC102549726 was evaluated and compared with the NC group by real-time PCR, and Si-435 was selected for subsequent experiments. n = 3 in each group. Values are expressed as mean ± SD. ^*^p < 0.05, vs. NC group, using an ANOVA test. (**b**) Knockdown of LOC102549726 by siRNA reversed the ISO-induced increase in mRNA levels of ANP, ATF 6 induced by ISO in NRCMs. n = 3 in each group. Values are mean ± SD. ^*^p < 0.05, vs. control group; ^#^p < 0.05, vs. ISO + NC group, respectively using Student’s t-test and one way ANOVA test. (**c,d**) Representative H&E staining images and quantitative analyses of myocardial hypertrophy based on cell surface area in the indicated groups. The hypertrophic response was reversed after transfection with si-LOC102549726 as compared to ISO-treated and negative control (NC) transfected groups. The datas are represented as mean ± SD, n = 3 for each group. Scale bar = 100 μm. ^*^p < 0.05 vs. control group; ^#^p < 0.05 vs. ISO + NC group, respectively using Student’s t-test and one way ANOVA test. (**e,f**) Representative immunoblots and quantitative analyses of ATF4, ATF6, eIF2α, and p-eIF2α in the indicated groups. si-LOC102549726 treatment significantly reduced ISO-induced overexpression of ATF6, eIF2α and p-eIF2α in NRCMs. Bars show the mean values ± SD. n = 3, ^*^p < 0.05 vs. control group; ^#^p < 0.05 vs. ISO + NC group, respectively using Student’s t-test and one way ANOVA test. (**g,h**) Representative immunoblots and quantitative analyses of p-IRE, XBP-1, HERG, SERCA2 and BNP in the indicated groups. si-LOC102549726 treatment enhanced the expression levels of HERG and SERCA2 than those in the control group, whereas the levels of p-IRE, XBP-1 and BNP reduced. Bars show the mean values ± SD. n = 3, ^*^p < 0.05 vs. control group; ^#^p < 0.05 vs. ISO + NC group, respectively using Student’s t-test and one way ANOVA test

To determine the role of LOC102549726 in CH, we downregulated LOC102549726 levels in NRCMs using siRNA. Real-time PCR (RT-PCR) was used to evaluate the interference efficiency of previously synthesized siRNAs. SiR-LOC102549726-435 was confirmed and selected for subsequent investigations (Fig. 3A). As displayed in Fig. 3B, the mRNA level of the hypertrophic marker ANP was significantly decreased in the si-LOC102549726 group compared to that in vector-treated NRCMs. H&E staining revealed that the hypertrophic response was reversed after transfection with si-LOC102549726 as compared to ISO-treated and negative control (NC) transfected groups, as evidenced by decreased cell surface area (Fig. 3 C and D). Subsequently, Western blot analysis demonstrated that treatment with si-LOC102549726 significantly reduced ISO-induced overexpression of ATF6, eIF2α, p-eIF2α, p-IRE, XBP1 and BNP in NRCMs. In contrast, HERG and SERCA2 protein levels were significantly higher than those in the control group (Fig. 3E-H). These results indicate that LOC102549726 aggravates ISO-induced CH and may act as a negative regulator of ER stress.

### Mir-760-3p involvement in the progression of cardiac hypertrophy

**Fig. 4:**
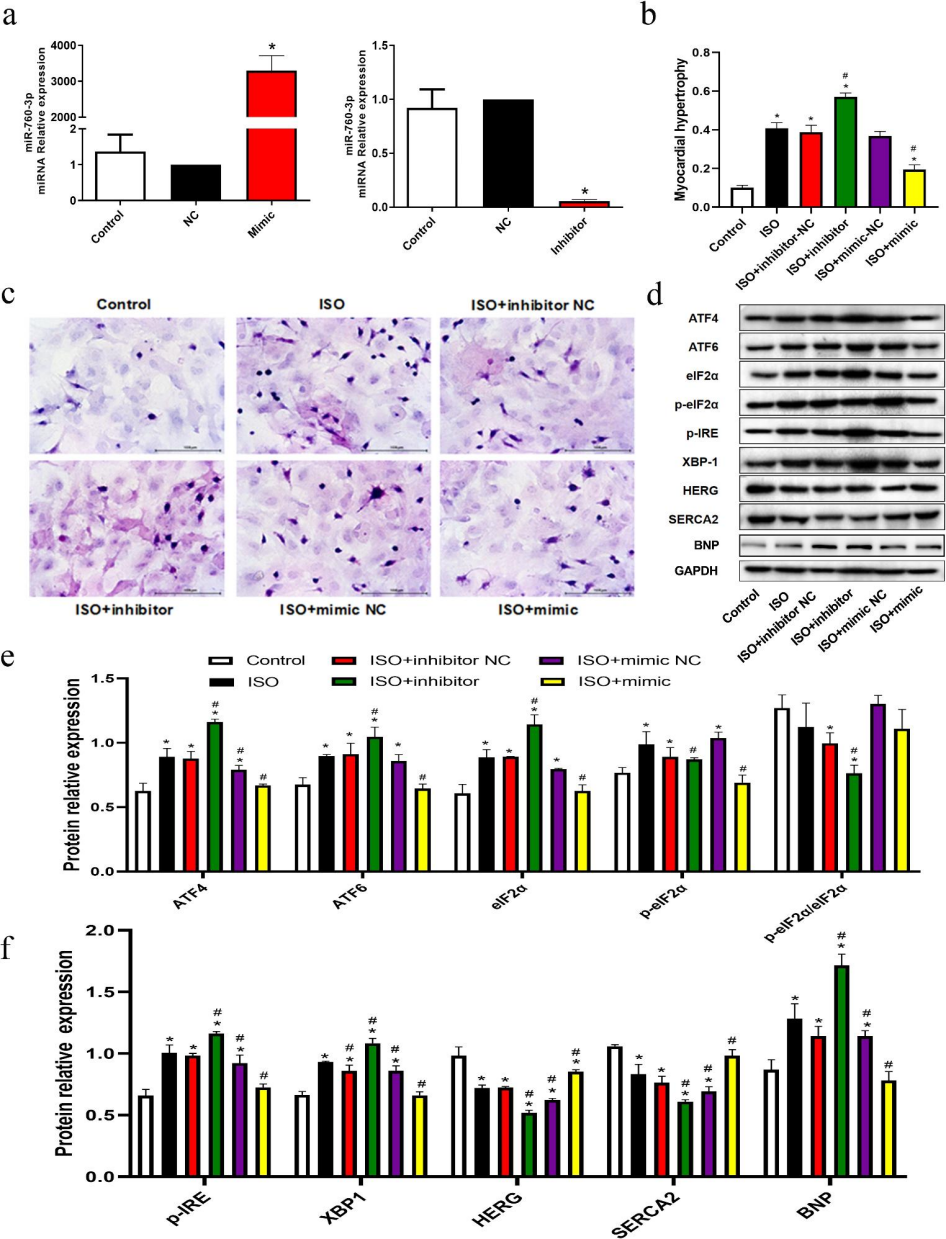
Roles of miR-760-3p in mediating the anti-hypertrophic effect in ISO-induced cardiomyocyte hypertrophy via ER stress. (**a**) The miRNA relative expression level of synthesized miR-760-3p mimic and miR-760-3p inhibitor were verified using qPCR. n = 3 in each group. Values are mean ± SD. ^*^p < 0.05, vs. NC group, using an ANOVA test. (**b,c**) Representative H&E staining images of cardiomyocytes and quantitative analyses of myocardial hypertrophy based on cell surface area in the indicated groups. Transfection with miR-760-3p inhibitor resulted in larger cell size than NC transfection, whereas miR-760-3p mimic significantly decreased cell surface area in NRCMs. The datas are represented as mean ± SD, n = 3 for each group. Scale bar = 100 μm. ^*^p < 0.05 vs. control group; ^#^p < 0.05 vs. ISO + NC group, respectively using Student’s t-test and one way ANOVA test. (**d-f**) Representative immunoblots and quantitative analyses of ATF4, ATF6, eIF2α, p-eIF2α, p-IRE, XBP-1, HERG, SERCA2 and BNP in NRCMs transfected with miR-760-3p mimic and inhibitor upon ISO treatment in the indicated groups. MiR-760-3p inhibitor enhanced the expression of ATF4, ATF6, eIF2α, p-eIF2α, p-IRE, XBP-1 and BNP, while simultaneously reducing p-eIF2α, p-eIF2α/eIF2α ratio, HERG, and SERCA2. Conversely, transfection with miR-760-3p mimic showed the opposite effect. Bars show the mean values ± SD. n = 3, *p < 0.05 vs. control group; ^#^p < 0.05 vs. ISO + NC group, respectively using Student’s t-test and one way ANOVA test

To further investigate the regulatory effect of miR-760-3p in ISO-induced CH, we transfected ISO-induced hypertrophic cardiomyocytes with miR-760-3p mimic, miR-760-3p inhibitor, and matched controls. RT-PCR analysis confirmed that the expression level of miR-760-3p was successfully up-regulated and downregulated (Fig. 4A). H&E staining revealed that miR-760-3p inhibitor transfection resulted in larger cell size than NC transfection, whereas miR-760-3p mimic treatment significantly decreased cell surface area in NRCMs (Fig. 4B and C). Furthermore, after transfecting ISO-pre-treated NRCMs with the miR-760-3p inhibitor, the expression of ATF4, ATF6, eIF2α, p-eIF2α, p-IRE, XBP-1 and BNP was dramatically enhanced, accompanied by a significant decrease in p-eIF2α, p-eIF2α/eIF2α ratio, HERG, and SERCA2. In contrast, transfection of miR-760-3p mimic into ISO-induced hypertrophic cardiomyocytes lowered the expression of the ER stress sensors ATF4, ATF6, eIF2α, p-eIF2α, p-IRE, XBP-1 and BNP. However, HERG and SERCA2 levels were significantly elevated compared with those in vector-treated cardiomyocytes (Fig. 4D-F). These results revealed that miR-760-3p is a critical regulator that participates in ISO-induced CH and that ER stress sensors may act as downstream targets.

## Discussion

Cardiovascular diseases are a major health issue and the leading cause of death worldwide (Nakamura and Sadoshima 2018). CH, fibrosis, and ventricular remodeling are the main pathological features of cardiovascular diseases (Liu et al. 2020). Pathological CH is manifested by cardiomyocyte enlargement and cardiac malfunction (Shimizu and Minamino 2016). Consequently, identifying key regulators of pathological CH is of great value for developing new therapeutic strategies. According to a previous study, the LOC102549726/miR-760-3p/Atf6 network was up-regulated in Ni-induced steroid synthesis disorder in rat Leydig cells (E et al. 2020). In the present study, the functional role of the LOC102549726/miR-760-3p network in the ISO-induced cardiomyocyte hypertrophy model was further explored. Specifically, we found that LOC102549726 was positively correlated with CH, which was further demonstrated in vitro using siRNA transfection. Next, we determined the level of miR-760-3p, which was significantly reduced in CH. Upregulation of miR-760-3p significantly attenuated the cell surface area and enhanced hypertrophy, and vice versa. In addition, we elucidated the potential mechanism of the LOC102549726/miR-760-3p network during in vitro ISO stimulation. Western blotting and RT-qPCR assays revealed that ER stress markers ATF4, ATF6, eIF2α, p-eIF2α, p-IRE, and XBP-1 were significantly altered, followed by LOC102549726/miR-760-3p regulation, as well as potassium and calcium channels. Taken together, these findings may provide a new direction for developing therapeutic targets for pathological CH.

Non-coding RNAs, such as lncRNAs and miRNAs, are emerging as important regulators of diverse pathological processes (Costa et al. 2018). Typically, non-coding RNAs exert their function by interacting with regulatory proteins (Panni et al. 2020). Recently, lncRNA has attracted widespread interest and has been explored concerning many diseases. Myosin heavy chain-associated RNA transcript (MHRT), a cardiac-specific lncRNA, has been depicted to protect the heart against pathological CH (Xu et al. 2020). LncRNA cardiac-hypertrophy-associated-transcript (CHAST) promotes hypertrophy by disrupting beneficial autophagic processes via down-regulation of Plekhm1 (Viereck et al. 2016). lncRNA HOTAIR functions as a negative regulator of CH (Lai et al. 2017). Following these studies, we found that the level of LOC102549726 significantly increased in ISO-induced hypertrophic cardiomyocytes. In addition, the downregulation of LOC102549726 attenuated hypertrophy, as evidenced by the decreased enlarged cell surface area and enhanced mRNA and protein levels of hypertrophic markers (Fig. 3). Additionally, it is widely known that miRNAs primarily act by inhibiting gene expression, leading to the degradation of targeted mRNAs. Previous studies displayed that miR-760-3p plays a vital role in the OGD/R cell model (Zhang et al. 2022). In the present investigation, miR-760-3p levels were lowered in hypertrophic cardiomyocytes. In vitro inhibition of miR-760-3p by miRNA inhibitors aggravates CH. In contrast, transfection of the miR-760-3p mimic into cardiomyocytes abolished CH (Fig. 4). These results revealed for the first time that the LOC102549726/miR-760-3p network contributes to CH progression.

The ER stress signaling pathway focused on elucidating the potential mechanism of LOC102549726/miR-760-3p network-associated modulation in CH under ISO treatment. Previous studies have confirmed that ER stress plays a central role in the development of cardiovascular diseases and that the UPR is implicated in the stress response during CH progression (Yao et al. 2017). The stimulator of interferon genes (STING) was validated as a critical regulator for pathological CH, predominantly mediated by ER stress (Zhang et al. 2020b). Another study demonstrated that ER stress markers, including eukaryotic translation initiation factor 2 submit alpha (eIF2α), IRE-1, and C/EBP homologous protein (CHOP), were significantly elevated in a transverse aortic constriction (TAC) surgery mouse model (Zhang et al. 2019). Consistent with previous studies (Liu et al. 2021), we found that the levels of p-eIF2α, p-IRE, and X-box binding protein 1 (XBP-1) were significantly increased in hypertrophic NRCMs. Downregulation of LOC102549726 resulted in decreased expression of ATF6, eIF2α, p-eIF2α, p-IRE, and XBP-1. However, transfection with miR-760-3p inhibitor increased the levels of ATF4, ATF6, eIF2α, p-eIF2α, p-IRE, and XBP-1. In contrast, miR-760-3p mimic treatment had the opposite effect. Consequently, we hypothesized that the LOC102549726/miR-760-3p network mediates CH through ER stress.

Similarly, ion dysregulation is an important factor in inducing ER stress (Binas et al. 2020). Increasing evidence suggests that ion levels, especially calcium and potassium levels, contribute to the development of CH. Elevated Ca^2+^ levels trigger the CH response in primary cardiomyocytes (Zhang et al. 2010). Decreased K^+^ current densities and impaired repolarization lead to QT prolongation and are closely associated with CH (Yang et al. 2012). K^+^ currents, and channel transcripts were significantly decreased in a transgenic dilated cardiomyopathy model accompanied by prolonged QT intervals (Yang et al. 2010). Our in vitro experiments demonstrated that the sarcoendoplasmic reticulum (SR) calcium transport ATPase 2 (SERCA2), a protein that can pump free Ca^2+^ ions into the ER lumen (Primeau et al. 2018), was markedly increased, followed by LOC102549726 interfering with miR-760-3p mimic treatment.

In contrast, treatment with miR-760-3p inhibitor markedly attenuated ISO-induced SERCA2 expression. The human ether-à-go-go-related potassium channel (hERG), which encodes rapidly activating delayed rectifying potassium current (IKr) (Vandenberg et al. 2012), has a consistent result with SERCA2 after transfection with si-LOC102549726 and miR-760-3p, respectively. These results indicate that a complicated relationship exists between the LOC102549726/miR-760-3p network and the ER stress response and that ions such as Ca^2+^ and K^+^ may serve as mediators in the development of CH.

In the present study, we revealed that the LOC102549726/miR-760-3p network is involved in pathological CH via ER stress. The direct interaction between LOC102549726 and miR-760-3p remains unproven; therefore, a dual luciferase reporter gene assay may be required in the future. Additionally, only primary NRCMs were used in this study and an in vivo experiment was performed.

**Fig. 5:**
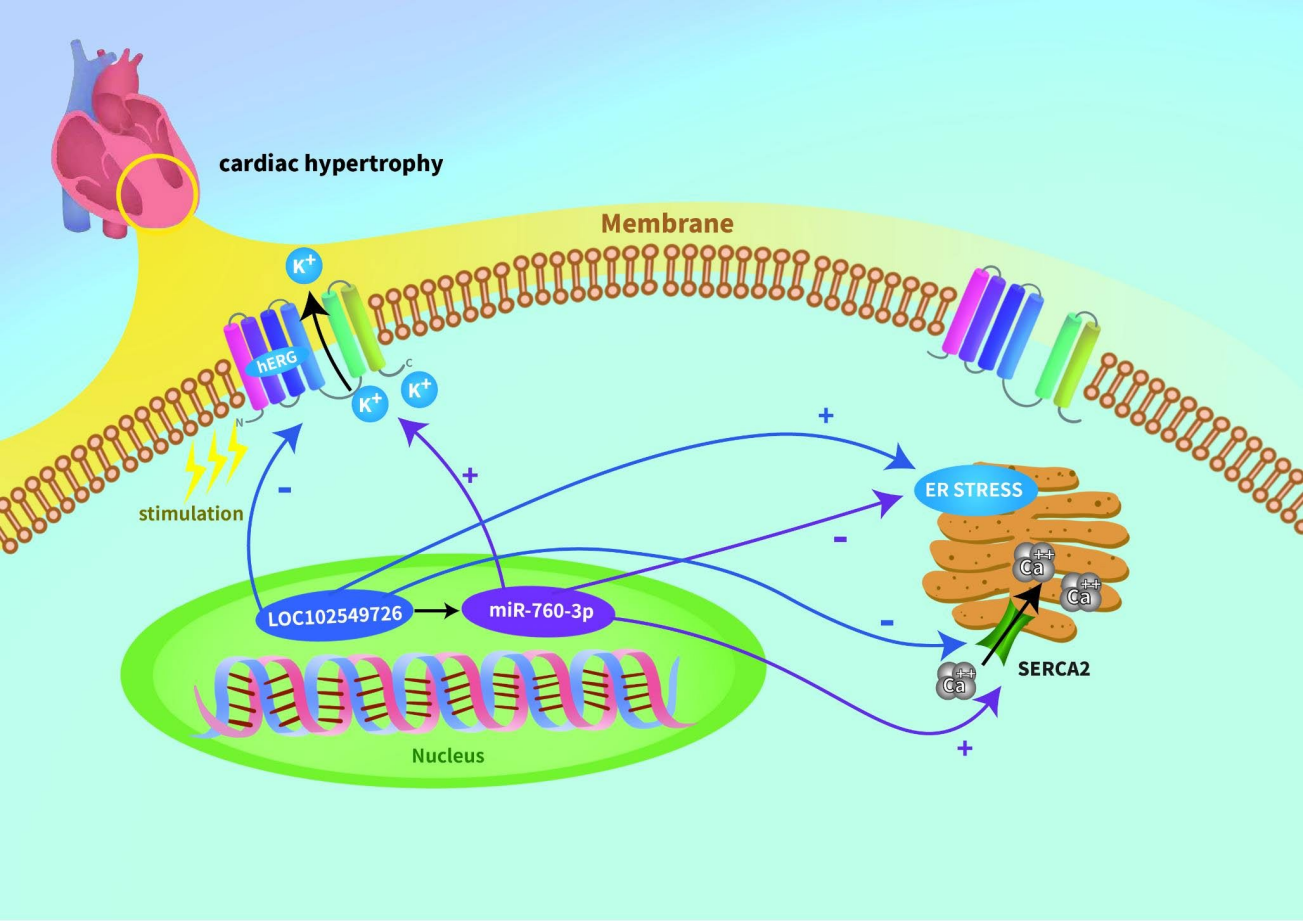
Schematic cartoon showing the proposed mechanism linking LOC102549726/miR-760-3p network, ER stress response, and ion channels to CH. lncRNA LOC102549726 is up-regulated in pathological CH. Downregulation of LOC102549726 mitigated its action on hypertrophy, accompanied by a decreased ER stress response and restored ion channel function. However, the downregulation of miR-760-3p deteriorates CH and enhances ER stress, thereby repressing the channel function. In contrast, the upregulation of miR-760-3p has a beneficial effect on ISO-induced cardiomyocyte hypertrophy. These results reveal an unexplored lncRNA/miRNA network associated with downstream targets that function in CH

In conclusion, the present study revealed the crucial role of the LOC102549726/miR-760-3p network in ISO-induced CH. In vitro experiments further explored the underlying mechanism by which the LOC102549726/miR-760-3p network mediates CH through ER stress and the ions that may participate in this process (Fig. 5). Thus, our study improves the understanding of the molecular mechanisms underlying pathological CH and develops novel therapeutic strategies for treating CH.

## Data Availability

The authors confirm that the data supporting the findings of this study are available within the article.

## Abbreviations

β-MHC: β-myosin heavy chain
ANP: Atrial natriuretic peptide
ATF4: Activating transcription factor 4
ATF6: Activating transcription factor 6
BNP: Brain natriuretic peptide
ceRNA: Competing endogenous RNA
eIF2α: Eukaryotic initiation factor 2α
ER stress: Endoplasmic reticulum stress
HE: Hematoxylin and eosin
HERG: Potassium voltage-gated channel subfamily H member 2
ISO: Isoproterenol
LncRNA: Long non-coding RNA
mRNA: Messenger RNA
NC: Negative control
NRCMs: Primary neonatal rat cardiomyocytes
p-eIF2α: Phosphorylation of eukaryotic initiation factor 2α
p-IRE: Phosphorylation of inositol requiring enzyme
PCR: Polymerase chain reaction
SERCA2: Sarcoendoplasmic reticulum (SR) calcium transport ATPase 2
siRNA: Small interfering RNA
XBP-1: X box binding protein 1
